# Intra-host variation and evolutionary dynamics of SARS-CoV-2 populations in COVID-19 patients

**DOI:** 10.1186/s13073-021-00847-5

**Published:** 2021-02-22

**Authors:** Yanqun Wang, Daxi Wang, Lu Zhang, Wanying Sun, Zhaoyong Zhang, Weijun Chen, Airu Zhu, Yongbo Huang, Fei Xiao, Jinxiu Yao, Mian Gan, Fang Li, Ling Luo, Xiaofang Huang, Yanjun Zhang, Sook-san Wong, Xinyi Cheng, Jingkai Ji, Zhihua Ou, Minfeng Xiao, Min Li, Jiandong Li, Peidi Ren, Ziqing Deng, Huanzi Zhong, Xun Xu, Tie Song, Chris Ka Pun Mok, Malik Peiris, Nanshan Zhong, Jingxian Zhao, Yimin Li, Junhua Li, Jincun Zhao

**Affiliations:** 1 State Key Laboratory of Respiratory Disease, National Clinical Research Center for Respiratory Disease, Guangzhou Institute of Respiratory Health, The First Affiliated Hospital of Guangzhou Medical University, Guangzhou, 510120 Guangdong China; 2 BGI-Shenzhen, Shenzhen, 518083 China; 3 Shenzhen Key Laboratory of Unknown Pathogen Identification, BGI-Shenzhen, Shenzhen, 518083 China; 4 Institute of Infectious Disease, Guangzhou Eighth People’s Hospital of Guangzhou Medical University, Guangzhou, 510060 Guangdong China; 5 BGI Education Center, University of Chinese Academy of Sciences, Shenzhen, 518083 China; 6 BGI PathoGenesis Pharmaceutical Technology Co., Ltd, BGI-Shenzhen, Shenzhen, 518083 China; 7 Department of Infectious Diseases, Guangdong Provincial Key Laboratory of Biomedical Imaging, Guangdong Provincial Engineering Research Center of Molecular Imaging, The Fifth Affiliated Hospital, Sun Yat-sen University, Zhuhai, 519000 Guangdong China; 8 Yangjiang People’s Hospital, Yangjiang, Guangdong China; 9 School of Biology and Biological Engineering, South China University of Technology, Guangzhou, China; 10 School of Future Technology, University of Chinese Academy of Sciences, Beijing, 101408 China; 11 Guangdong Provincial Key Laboratory of Genome Read and Write, BGI-Shenzhen, Shenzhen, 518120 China; 12 Guangdong Provincial Center for Disease Control and Prevention, Guangzhou, 511430 Guangdong China; 13 The HKU–Pasteur Research Pole, School of Public Health, Li Ka Shing Faculty of Medicine, The University of Hong Kong, Hong Kong, SAR, 19406 China

**Keywords:** SARS-CoV-2, COVID-19, Intra-host, Variation, Dynamics

## Abstract

**Background:**

Since early February 2021, the causative agent of COVID-19, SARS-CoV-2, has infected over 104 million people with more than 2 million deaths according to official reports. The key to understanding the biology and virus-host interactions of SARS-CoV-2 requires the knowledge of mutation and evolution of this virus at both inter- and intra-host levels. However, despite quite a few polymorphic sites identified among SARS-CoV-2 populations, intra-host variant spectra and their evolutionary dynamics remain mostly unknown.

**Methods:**

Using high-throughput sequencing of metatranscriptomic and hybrid captured libraries, we characterized consensus genomes and intra-host single nucleotide variations (iSNVs) of serial samples collected from eight patients with COVID-19. The distribution of iSNVs along the SARS-CoV-2 genome was analyzed and co-occurring iSNVs among COVID-19 patients were identified. We also compared the evolutionary dynamics of SARS-CoV-2 population in the respiratory tract (RT) and gastrointestinal tract (GIT).

**Results:**

The 32 consensus genomes revealed the co-existence of different genotypes within the same patient. We further identified 40 intra-host single nucleotide variants (iSNVs). Most (30/40) iSNVs presented in a single patient, while ten iSNVs were found in at least two patients or identical to consensus variants. Comparing allele frequencies of the iSNVs revealed a clear genetic differentiation between intra-host populations from the respiratory tract (RT) and gastrointestinal tract (GIT), mostly driven by bottleneck events during intra-host migrations. Compared to RT populations, the GIT populations showed a better maintenance and rapid development of viral genetic diversity following the suspected intra-host bottlenecks.

**Conclusions:**

Our findings here illustrate the intra-host bottlenecks and evolutionary dynamics of SARS-CoV-2 in different anatomic sites and may provide new insights to understand the virus-host interactions of coronaviruses and other RNA viruses.

**Supplementary Information:**

The online version contains supplementary material available at 10.1186/s13073-021-00847-5.

## Background

In December 2019, a new coronavirus, severe acute respiratory syndrome coronavirus 2 (SARS-CoV-2), started an outbreak of pneumonia infections in Wuhan, Hubei Province, China. SARS-CoV-2 represents efficient infectivity and transmissibility. It transmits efficiently among human beings, with an R0 estimated to be over 2 [1, 2]. Most symptomatic patients infected by SARS-CoV-2 display symptoms of fever, cough, fatigue, myalgia, dyspnea, or pneumonia [3]. Moreover, infected individuals without apparent clinical symptoms may be also able to transmit the viruses to their contacts [4]. It is of great concern to us all as to why such a newly emerging virus could spread in human populations so rapidly, which urges the investigation into the origin, virus-host interactions, and evolutionary pathway of SARS-CoV-2.

The novel coronavirus is now causing a global pandemic which has severely impacted health care systems, economies, and societies worldwide. Understanding of mutation and evolution within the intra-host and inter-host populations of SARS-CoV-2 provides important information on transmission and pathogenesis of this virus. RNA virus replication typically has a high error rate than DNA viruses due to the lack of sufficient proofreading activities during genome replication [5, 6]. As the largest and most complex known RNA virus genomes, CoV genomes employ nsp14 to enhance the fidelity of RNA synthesis, which is highly conserved and has the exonuclease proofreading function. Since the outbreak of COVID-19, SARS-CoV-2 has rapidly spread around the world with an estimated evolutionary rate of (8–9) × 10^−4^ nucleotide substitutions per site per year [7, 8], which is similar with previously reported rates of SARS-CoV (8.0–23.8 × 10^−4^) [9] and MERS-CoV (6.3–11.2 × 10^−4^) [10, 11]. During the first several months of the COVID-19 pandemic, multiple genotypes (S, L, V, G, GH, GR, and O) [12–14] and hundreds of SNPs scattered throughout the genome have been reported [15–18]. Although plenty of polymorphic sites have been identified among SARS-CoV-2 populations, the true variant spectra of closely related viral genomes within the same host are mostly disguised by the consensus sequence [19]. Intra-host variant spectra of closely related viral genomes remain largely unknown.

Previous studies on Ebola [20], influenza virus [21], and yellow fever virus (YFV) [22] have shown that iSNVs that appeared during the course of the epidemic could provide valuable information about the size of transmission bottleneck, human-to-human transmission chain, and viral diversity. For instance, influenza A virus (IAV) and influenza B virus (IBV) transmission bottlenecks are both stringent [23], while IBV exhibits lower intra-host diversity compared to IAV [21]. This pattern of intra-host viral evolution is consistent with influenza B virus’ slower global evolutionary rate. Comparison of the YFV evolutionary rates estimated by iSNV and SNP indicated that the intra-host evolutionary rate was much higher than that during the epidemic [22], which reflected purifying selection occurred. As for the novel coronavirus SARS-CoV-2, the latest research based on three COVID-19 patients indicated that SARS-CoV-2 exhibits intra-host genomic plasticity and low-frequency polymorphic quasispecies [24–26]. Understanding the full underlying intra-host diversity is closely related to transmission pattern and vaccine design. Of particular, the main route of SARS-CoV-2 shedding is via both the respiratory tract (RT: nose swab, sputum, throat swab) [27] and gastrointestinal tract (GIT: anus-anal swab, feces) samples [28, 29]; the intra-host diversity and characteristics of SARS-CoV-2 in different anatomic sites have not been addressed.

Here, using high-throughput sequencing of metatranscriptomic and hybrid captured libraries, we characterized consensus genomes and intra-host variations of serial samples collected from eight patients with COVID-19. The frequency of mutated allele frequencies changes dramatically among either sample types or time points. Specifically, we observed a remarkable differentiation between SARS-CoV-2 populations of fecal samples and that of the respiratory tract samples within the same patient. The genomic resources presented here are of importance for the research community to estimate human-to-human transmission and to understand the intra-host evolutionary dynamics of SARS-CoV-2 population in patients with COVID-19. The findings of the present study also shed light on the prevention and control of SARS-CoV-2.

## Methods

### Patient enrolment

Eight pneumonia patients, referred as the GZMU cohort, were confirmed with SARS-CoV-2 infection between January 25 and February 10 in 2020 and hospitalized at the first affiliated hospital of Guangzhou Medical University (six patients), the fifth affiliated hospital of Sun Yat-sen University (one patient), and Yangjiang People’s Hospital (one patient). Serial samples were collected, including nasal swabs, throat swabs, sputum, gastric mucosa, urine, plasma, anal swabs, and feces. All the information regarding patients has been anonymized.

### Real-time RT-qPCR and metatranscriptomic sequencing

A total of 62 serial clinical samples collected from eight patients with COVID-19 (Additional file 1: Table S1) were used for real-time RT-qPCR. Clinical samples were subjected to RNA extraction using QIAamp Viral RNA Mini Kit (Qiagen, Hilden, Germany). An in-house real-time RT-qPCR was performed by targeting the SARS-CoV-2 RdRp and N gene regions (Zybio Inc.). Human DNA was removed using DNase I and RNA concentration was measured using Qubit RNA HS Assay Kit (Thermo Fisher Scientific, Waltham, MA, USA). DNA-depleted and purified RNA was used to construct double-stranded (ds) cDNA library using MGIEasy RNA Library preparation reagent set (MGI, Shenzhen, China) following the protocol described in our previous study [30]. Specifically, the ds cDNA was Unique Dual Indexed to increase sequencing specificity. To track possible contamination, human breast cell lines (Michigan Cancer Foundation-7) were used as controls during library construction. High-throughput sequencing of the constructed libraries was then carried out on the DNBSEQ-T7 platform (MGI, Shenzhen, China) to generate metatranscriptomic data of 100-bp paired-end reads.

### Hybrid capture-based enrichment and sequencing

For a subset of samples (Additional file 1: Table S1), genomic content of SARS-CoV-2 was enriched from the double-stranded cDNA libraries mentioned above using the 2019-nCoVirus DNA/RNA Capture Panel (BOKE, Jiangsu, China) as described in our previous study [30]. In detail, negative controls were prepared using the total RNA from MCF-7 breast cancer cell and nuclease-free water. According to the instruction of MGISEQ-2000 platform, the SARS-CoV-2 content-enriched samples were used to construct DNA Nanoballs (DNBs)-based libraries and sequenced on the MGISEQ-2000 platform to generate data of 100-bp paired-end reads. Data extraction and cleaning were performed prior to analysis.

### Data filtering and genome assembly

Data filtering was performed following the procedures described in previous research [30]. Briefly, for both metatranscriptomic and hybrid capture data, sequence data of each sample were firstly mapped to a pre-defined database comprising representative genomes of coronaviridae. The mapped reads were then subject to the removal of low-quality, duplications, adaptor contaminations, and low-complexity to collect high-quality coronaviridae-like reads. We also compared the allele frequencies among the two data types (metatranscriptomic sequencing and hybrid capture-based sequencing methods) when available; samples with conflicted consensus alleles were removed. For the samples with 60-fold of metatranscriptomic data, coronaviridae-like metatranscriptomic reads were used to generate consensus genomes and identify intra-host variants. Full-length consensus genomes were generated from reads mapped to the reference genome (GISAID accession: EPI_ISL_402125) using Pilon (v. 1.23) [31]. To prevent false discovery, base positions reporting an alternative allele with the following conditions were masked as N: (1) sequencing coverage less than 5-fold and (2) sequencing coverage less than 10-fold and the proportion of reads with the alternative allele less than 80%. The collected coronaviridae-like reads were also de novo assembled using SPAdes (v. 3.14.0) with default settings [32] with a maximum of 100-fold coverage of read data. Structural variations between the de novo assemblies and consensus genomes, if any, were manually checked and resolved based on read alignments. Nucleotide differences between the consensus sequences and the reference genome were summarized into artificial Variant Call Format (VCF) files, which were annotated using SnpEff (v.2.0.5) [33] with default settings.

### Phylogenetic analysis

Available consensus sequences of SARS-CoV-2 (Additional file 1: Table S2) were collected from GISAID database (https://www.gisaid.org/) on 5 April 2020, after the removal of highly homologous sequences, 122 representative virus strains (Additional file 1: Table S2) were used to infer evolutionary relationships with the assembled genomes. Within the GZMU cohort, only one genome was selected when more than one identical genome was achieved from the same patient. The assembled SARS-CoV-2 and selected representative genomes were aligned using MAFFT with default settings. A maximum likelihood (ML) tree was inferred using the software IQ-TREE (v.1.6.12) [34], with the best fit nucleotide substitution model selected by ModelFinder from the same software. The inferred ML tree was then visualized using the R package ggtree [35] (v.3.10). Major branches and the defining nucleotide mutations were manually labeled.

### Summary of public consensus variants

All the consensus sequences of the public strains were aligned with the reference genome (GISAID accession: EPI_ISL_402125) using MAFFT (v.5.3) [36] with default settings. Nucleotide differences between the consensus sequences and the reference genome were summarized into an artificial VCF file, which was then were annotated using SnpEff (v.2.0.5) with default settings. The linkage disequilibrium among the identified consensus variants was estimated using VCFtools (v.0.1.16).

### Calling of iSNVs

Here, an intra-host single nucleotide variant (iSNV) was defined as the alternative allele co-existed with the reference allele at identical genomic position within the same sample. To minimize false discovery, iSNVs were identified on samples with at least 60-fold mean metatranscriptomic sequencing coverage and then verified using hybrid capture data when available.

First, paired-end metatranscriptomic reads were mapped to the reference genome (GISAID accession: EPI_ISL_402125) using BWA aln (v.0.7.16) with default parameters [37]. Duplicated reads were marked using Picard MarkDuplicates (v. 2.10.10) (http://broadinstitute.github.io/picard) with default settings. Base composition of each position was summarized from the mapped reads using the software pysamstats (v. 1.1.2) (https://github.com/alimanfoo/pysamstats), and then subject to iSNV site identification with the following criteria: (1) base quality larger than 20, (2) sequencing coverage of paired-end mapped reads ≥ 10, (3) at least five reads support the minor allele, (4) minor allele frequency ≥ 5%, and (5) strand bias ratio of reads with the minor allele and reads with major allele less than ten-fold. To minimize false discoveries, sites with more than one alternative allele were filtered out. Biological effects of the identified iSNVs were annotated using the SnpEff (v.2.0.5) with default settings. Alternative allele frequencies (AAFs) at the identified iSNV sites were measured by the proportion of paired-end mapped reads with alternative alleles. To avoid the bias caused by the large range of viral genome coverage, we only selected the samples with sufficient metatranscriptomic data (>60X) as described above. Most (26/32) of the 32 samples had >200X average sequencing coverage, allowing iSNV site to be confidently defined with a 5% allele frequency cut-off (which means at least 10 supporting reads). Given that most samples (27/32) were also hybrid capture sequenced, all the iSNVs of those samples were verified and supported by at least two hybrid capture reads from the same sample, showing that the iSNV identification is robust and solid. For the 26 samples with at least 200X data, most genomic regions were covered by at least 100X of read data, ensuring that the iSNVs with low frequencies (2~5%) could be confidently identified. In fact, all the iSNVs were identified with a depth > 50X (Additional file 1: Table S3). Furthermore, the detection cut-off of that iSNV was reduced to 2% for the rest of the samples of the same patient, when an iSNV was detected in one patient, only the AAFs more than 2% with at least three reads were kept for the following analyses. The accuracy of the iSNV detection is also reflected by the high concordance between the two pairs of replicates (Additional file 2: Fig. S1). The improved methodologies should ensure a confident identification of iSNVs among samples with a large range of coverage. All the iSNVs were verified using hybrid capture data when applicable. At the iSNV sites, the allele with higher frequency was defined as a major allele, while one with less frequency was defined as a minor allele, regardless whether it is different from the reference allele. A heatmap was generated to visualize the AAFs for all samples using the pheatmap package in R (v.3.6.1). A subset of the identified iSNVs were validated by Sanger sequencing using the protocol described in the previous study [30].

### Statistics of iSNVs

The distribution of iSNVs among genetic components and patients were summarized and visualized using the Python package matplotlib (v.3.2.1). Alternative allele frequencies on all the detected iSNV sites were compared among patients. To avoid oversampling, for the patients with more than one sample, only the median AAF among all samples of that patient was used for comparison. Alternative allele frequencies among synonymous and non-synonymous variants and among codon positions were compared using the Wilcoxon rank-sum test and visualized through box plot using the R package *ggplot* (v.3.3.0). For the iSNVs detected in patient P01 and P08, the dynamics of AAFs was visualized across time points using the R package *ggplot* (v.3.3.0).

### Genetic diversity

The genetic diversity of each sample was estimated using Shannon entropy based on the AAF of each iSNV, assuming that all iSNVs are independent from each other.

$$ H(x)=-\sum \limits_i^nP(i){\mathit{\log}}_2P(i) $$

where *P*(*i*) is the AAF at variable site *i*. The comparison of genetic diversities between RT and GIT samples was performed using the Wilcoxon rank-sum test.

### Genetic distance

The genetic distance among samples was estimated using L1-norm distance in a pairwise manner.

$$ D=\sum \limits_{k=1}^N\sum \limits_{i=1}^n\mid {p}_i-{q}_i\mid $$

The L1-norm distance (*D*) between a pair of samples is the sum of the distance across all the variable sites (*N*). For each variable site, the distance is calculated between vectors (*p* and *q* for each sample) comprising frequencies of all the four possible nucleotide bases (*n* = 4). The comparison of genetic distances among different categories of sample pairs was performed using the Wilcoxon rank-sum test.

### Haplotype reconstruction

Haplotypes of neighbor iSNV sites were reconstructed using mapped paired-end reads.

## Results

### Clinical characteristics of the patients with COVID-19

**Fig. 1:**
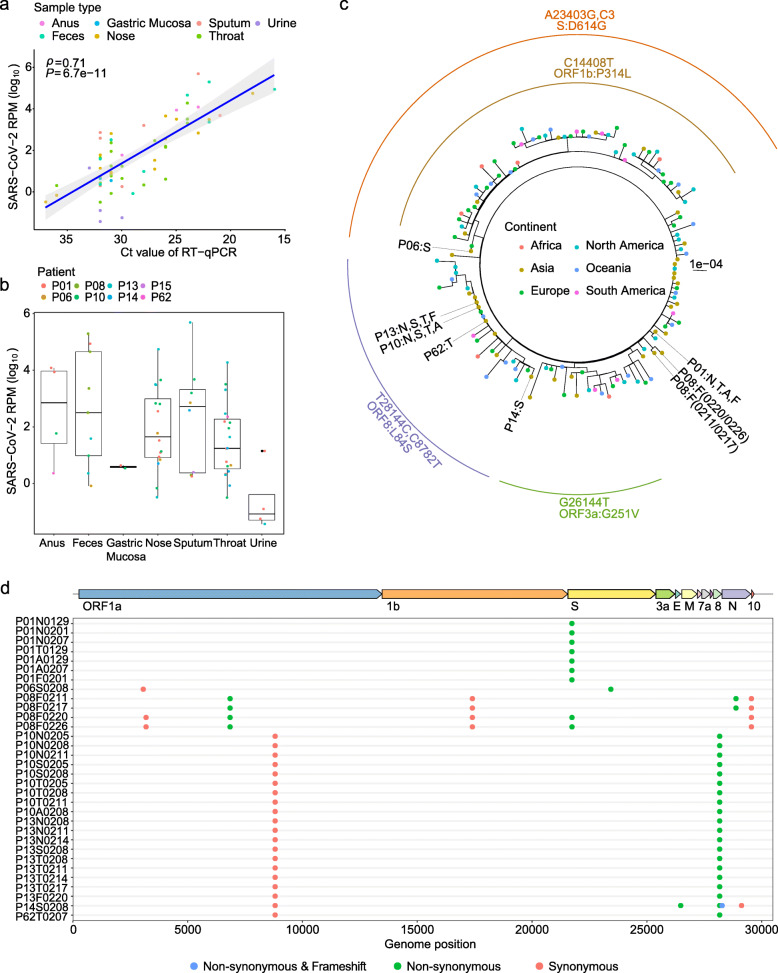
Sequence data from various sample types of patients with COVID-19. **a** SARS-CoV-2 RPM of metatranscriptomic data plotted against RT-qPCR cycle threshold (Ct) value for the clinical samples. **b** Frequency distribution of samples based on SARS-CoV-2 reads per million (SARS-CoV-2 RPM). **c** Maximum likelihood tree of consensus SARS-CoV-2 genomes using IQ-TREE. Colors of dotted tips represent the geographic locations of samples. Nucleotide mutations that define the branch were labeled outside the tree. **d** Distribution of consensus variants (in round circles) detected in the GZMU cohort across the SARS-CoV-2 genome. Colors represent the biological effect of mutations. Non-synonymous variants are denoted by green, synonymous variants by red, and frameshift by blue. EPI_ISL_402125 was used as the reference sequence

From January 25 to February 10 in 2020, we collected a total of 62 serial clinical samples from eight hospitalized patients (GZMU cohort) confirmed with SARS-CoV-2 infection using real-time RT-qPCR (Additional file 1: Table S1). All patients had direct contacts with confirmed cases during the early stage of the outbreak. Most patients, except P15 and P62, had severe symptoms and received mechanical ventilation in ICU, including patient P01 who passed away eventually. Patient P01 also showed much lower antibody (IgG and IgM) responses (Additional file 1: Table S1) compared to other patients. We then deep sequenced the 62 clinical samples using metatranscriptomic and/or hybrid capture methods (Additional file 1: Table S1). The numbers of SARS-CoV-2 reads per million (SARS-CoV-2 RPM) among the metatranscriptomic data correlated well with the corresponding RT-qPCR cycle threshold (Ct) of SARS-CoV-2, reflecting a robust estimation of viral load (*R* = 0.71, *P* = 6.7e−11) (Fig. 1a). The respiratory tract (RT: nose swab, sputum, throat swab) and gastrointestinal tract (GIT: anus-anal swab, feces) samples showed higher SARS-CoV-2 RPMs compared to gastric mucosa and urine samples (Fig. 1b). The data here may reflect an active replication of SARS-CoV-2 in RT and GIT, especially in patients with severe symptoms [38, 39].

### Consensus genomic variations

Here, using metatranscriptomic data, we obtained 32 consensus complete genomes from the clinical samples with at least 60-fold sequence coverage (Additional file 1: Table S1 and Table S4). Comparing the assemblies to the reference sequence (GISAID accession: EPI_ISL_402125) revealed 14 consensus variants (6 synonymous and 8 non-synonymous) located mostly in ORF1ab, S, ORF8, and N genes (Additional file 1: Table S4). Most of the consensus variants were also detected among public sequences, including the widespread associated variants (C8782T and T28144C) detected in four patients (P10, P13, P14, and P62). The novel consensus variant causes a frameshift at the end of ORF8 in patient P14, showing the phenotypic plasticity during the evolution and adaptation of SARS-CoV-2. Evolutionary relationships showed that the consensus SARS-CoV-2 genomes of the GZMU cohort belonged to distinct clades, including clades defined by T28144C and A23403G, respectively (Fig. 1c). Remarkably, we observed distinct SARS-CoV-2 genotypes co-existed in the GIT samples of patient (P08) with three nucleotide differences (Fig. 1d and Additional file 1: Table S4), suggesting independent replications of different SARS-CoV-2 genotypes within the same host [40]. The existences of different genotypes could be explained by co-transmission, recurring mutation, or alternative quasispecies developed from adaptive immune response; a similar phenomenon was reported in hepatitis C virus (HCV) [41] and human polyomavirus JC (JCV) [42].

### Detection and characteristics of iSNVs

**Fig. 2:**
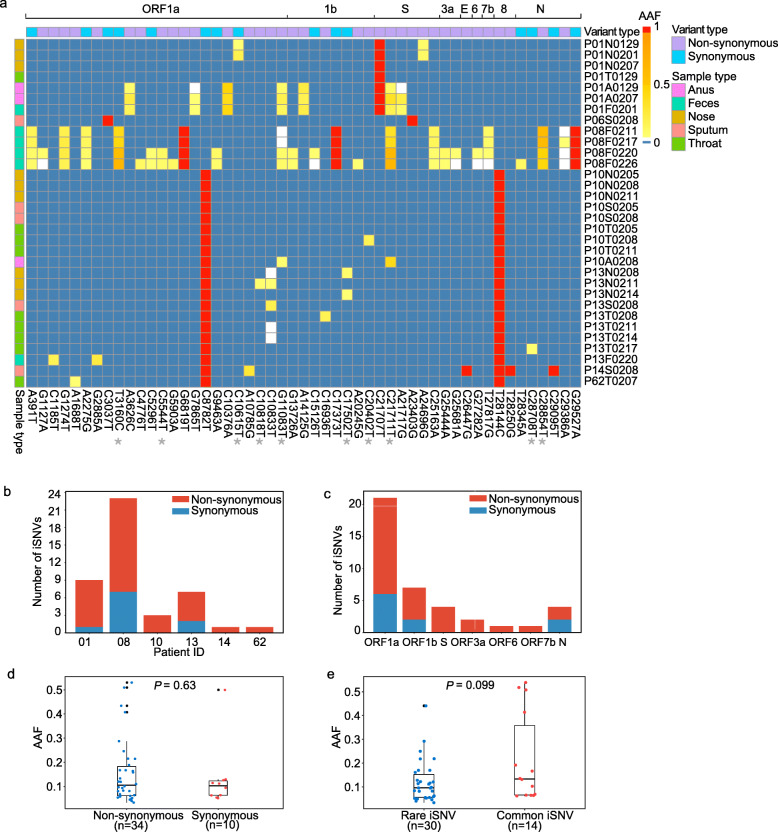
Characteristics of iSNVs. **a** Heatmap showing the alternative allele frequencies (AAFs) of intra-host single nucleotide variants (iSNVs) and consensus variants among samples. The sample (e.g., P01N0129) name indicates patient number P01, sample type (N nasal swab, T throat swab, A anal swab, F feces, S sputum) and collection date (January 29). Common iSNVs were marked by star symbols. Variant type and sample type were marked in different colors, and consensus variants were indicated in red. **b** The number of detected iSNVs per patient. **c** Number of iSNV sites among protein-encoding genes. **d** Box plot showing the distribution of alternative allele frequencies (AAFs) of non-synonymous and synonymous iSNVs. Each dot indicates the median AAF among all the detected iSNVs of samples from the same patient. **e** Box plot showing the distribution of AAFs of common and rare iSNVs. Each dot indicates the median AAF among all the detected iSNVs of samples from the same patient

Although plenty of polymorphic sites were identified among SARS-CoV-2 populations, intra-host variant spectra of closely related viral genomes are mostly disguised by the consensus sequences. We firstly examined the reproducibility of our experimental procedures for allele frequency estimation. Only a minor difference of alternative allele frequencies (AAFs) was observed among biological replicates of two selected samples (Additional file 2: Fig. S2), showing that the estimated population composition was marginally affected by independent experimental procedures. To control false discovery rate, we applied a stringent approach to detect iSNVs. The iSNVs were identified from the 32 samples using metatranscriptomic data and then verified using hybrid capture-based data, which are available for most (27/32) samples (Additional file 1: Table S5 and Table S3). Overall, we observed 1 to 23 iSNVs in six patients with a cut-off of 5% minor allele frequency (Fig. 2a, b). When an iSNV was discovered in one patient, we reduced the cut-off to 2% to detect that iSNV from the rest of the samples of the same patient (see the “Methods” section). The AAFs of iSNVs detected from the metatranscriptomic data correlated well with those of the hybrid capture-based data (Spearman’s *ρ* = 0.99, *P* < 2.2e−16; Additional file 2: Fig. S1). Furthermore, the numbers of the observed iSNVs did not correlate with the sequencing coverage (Additional file 2: Fig. S3), suggesting that the coverage of metatranscriptomic and hybrid capture-based data was sufficient to estimate intra-host variation in most samples.

We further analyzed intra-host variation across genes for evidence of purifying selection or neutral selection. If SARS-CoV-2 evolves under neutral selection, the ratio of non-synonymous substitution to synonymous substitution tends to be similar for all ORFs, and the iSNVs are likely to distribute equally at each codon position. In this research, the 40 identified iSNV sites (10 synonymous iSNVs and 30 non-synonymous iSNVs) distributed evenly across genomic regions (Fig. 2c; Additional file 1: Table S5). A similar number of iSNVs among codon positions (position 1: *n* = 15; position 2: *n* = 12; position 3: *n* = 13; chi-square test: *P* = 0.84) suggests that most iSNVs were under neutral selection or insufficient purifying selection. Meanwhile, we did not observe a significant difference in AAFs either between non-synonymous and synonymous iSNVs (Fig. 2d) or among codon positions (Additional file 2: Fig. S4), which also reflects the neutral selection of iSNVs.

### Co-occurring iSNVs

One central task when estimating intra-host variation is to identify the source of iSNVs. Overall, the distribution of the iSNVs among samples does not correlate well with the consensus SNPs (Fig. 2a). Samples carrying the same consensus SNPs generally had different iSNVs, particularly in P01, P10, and P13. Here, we classified the iSNVs into (i) rare iSNVs (30/40) detected in a single patient and (ii) common or shared iSNVs (10/40) detected in at least two patients and/or identical to consensus variants. The common iSNV could be used to estimate human-to-human transmission and the transmission bottleneck. Here, the ten common iSNVs did not show linkage with other consensus variants. Furthermore, their AAFs could not be discriminated from those of the rare iSNVs (Fig. 2e), reflecting a tight genetic bottleneck transmission. The comparison of SNPs and iSNVs also implies that most of the non-synonymous iSNVs could not be transmitted efficiently or the non-synonymous iSNVs might have disappeared, as most of them are deleterious. Notably, the common iSNVs include two iSNVs (G11083T and C21711T) exclusively detected in the GIT populations of P01, P08, and P10 (Additional file 1: Table S5). Among the common iSNVs, G11083T is the most widespread consensus variant distributed in multiple lineages of SARS-CoV-2, suggesting that it might be derived from recurring mutations on distinct strains rather than the mutation on a single ancestral strain. Furthermore, although G11083T was detected as an intra-host variant in the GIT samples of three patients, it was not detected in the corresponding RT samples, supporting that those loci might recurrently mutate. Interestingly, G11083T located in a region encoding a predicted T-cell epitope [43], suggesting that this suspected recurrent mutation may provide genetic plasticity to better adapt against host defenses. In addition, to exclude the possibility of potential sequencing errors influencing the accuracy of analysis, multiple sequencing methods, including metatranscriptomic and hybrid capture sequencing, were both performed with similar results obtained.

### Genetic diversity within the GIT and RT samples

**Fig. 3:**
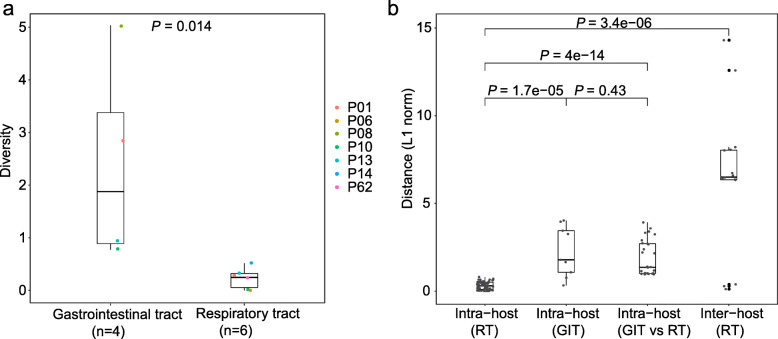
Genetic diversity and genetic distance. **a** Box plot showing the distribution of genetic diversity among samples from the gastrointestinal tract (GIT) and respiratory tract (RT). For the patients with more than one GIT/RT samples, only the median value was selected to represent the genetic diversity in GIT/RT. **b** Box plot showing the distribution of L1-norm distances among samples from the gastrointestinal tract (GIT) and respiratory tract (RT). Each dot represents the genetic distance between a unique pair

Using Shannon entropy, we observed a significantly higher genetic diversity within the GIT samples than that of RT samples (Wilcoxon rank-sum test, *P* = 1.4e−05; Fig. 3a and Additional file 1: Table S6), reflecting a larger viral population size within the GIT populations or under relaxed selective pressures. We further investigated the genetic differences between the two intra-host populations. Notably, no iSNV was shared between RT and GIT samples from the same patients, suggesting a frequent genetic differentiation among intra-host viral populations of different anatomic sites. Here, we used L1-norm distance to estimate genetic dissimilarity between sample pairs based on iSNVs and their AAFs (Fig. 3b and Additional file 1: Table S7). As expected, genetic distances among samples from the same host were smaller than those among inter-host samples (Fig. 3b and Additional file 1: Table S7). Within each host, the greatest genetic distance was observed among GIT samples and between GIT and RT samples, while the distances among RT samples were relatively small. For example, seven iSNVs were shared among the GIT samples of P01, while none of them was observed in RT samples (Fig. 2a). It seems that the clear genetic differentiation between GIT and RT populations is mostly driven by distant intra-host bottlenecks. However, the exact viral migration mechanisms among intra-host populations require further investigation.

### Allele frequency dynamics of iSNVs

**Fig. 4:**
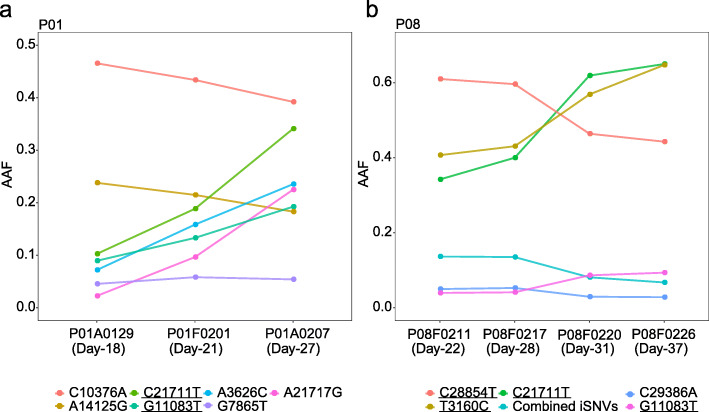
Temporal dynamics of intra-host populations in patients P01 and P08. **a**, **b** Alternative allele frequencies (AAFs) among sampling dates in patients P01 and P08. Days post the first symptom date are shown in the bracket. The sample (e.g., P01A0129) name indicates patient number P01, sample type (N nasal swab, T throat swab, A anal swab, F feces, S sputum), and collection date (January 29, 2020). Combined iSNVs are the average frequency of four similar iSNVs (A391T, A2275G, C25163A, and T27817G). Colors represent different iSNVs. Underlines represent common iSNVs

Previous studies have revealed longitudinal evolution of intra-host populations in some important RNA viruses [44–48]. We firstly compared the detected iSNVs among serial samples. All the iSNVs of early GIT samples also presented in later GIT samples, while most iSNVs detected in RT samples disappeared in at least one following sample, suggesting that the intra-host variants were better maintained in GIT. We further focused on the allele frequency dynamics of iSNVs observed in the GIT populations. Notably, most GIT iSNVs were remarkably stable and showed continuous trends of AAFs across sampling dates. For example, within the GIT population of P01, seven iSNVs showed continuous trends of allele frequency dynamics, including four iSNVs with increased AAFs and two iSNVs with decreased AAFs across the three sampling dates (Fig. 4a). Given their similar growth rates but distinct allele frequencies, it is likely that more than two genetically related haplotypes co-existed within the GIT population of P01. Similar patterns were also observed in the GIT population of P08 (Fig. 4b). Notably, the dynamics of intra-host variation simultaneously changed the consensus alleles (> 50%) of three viral genetic loci (3160, 21711, and 28854) in P08 (Fig. 2a), suggesting that polymorphisms on the three loci might correlate with each other. Despite those changes, GIT populations seemed to be relatively stable while developing towards a high genetic diversity. Nonetheless, in both P01 and P08, we observed increased AAFs of the GIT-specific variants (C21711T and G11083T). However, more evidence is required to examine whether tissue-specific adaptation actively involves in the divergence among distant intra-host populations.

### Development of intra-host diversity within the GIT population

We further phased proximal iSNVs into local haplotypes using paired-end mapped reads (Additional file 1: Table S8). Most minor haplotypes had one nucleotide difference from the dominant haplotype of the same sample, suggesting that they might be mutated from the main strain of the corresponding population. Nonetheless, we observed one exception in the GIT population of P01. On the variable loci of C21707T, C21711T, and A21717G, the GIT population of P01 showed one dominant haplotype (T-C-A) and two minor haplotypes (T-T-A and T-T-G) (Additional file 2: Fig. S5). Despite that one minor haplotype (T-T-A) was relatively stable (8–10%), the proportion of the dominant haplotype (T-C-A) decreased from 89 to 67%, while that of the other minor haplotype (T-T-G) increased from 2 to 22%. Based on the haplotype dynamics and their nucleotide differences, we hypothesized that the minor haplotype (T-T-G) may be derived from the dominant haplotype (T-C-A) via the intermediate minor haplotype (T-T-A), which was consistent with the recent report of widespread occurrence of C → U hypermutation in the genomes of SARS-CoV-2 [49]. Importantly, our observation supports that the mutated viruses further replicate and accumulate variants in the GIT population, and finally, evolve towards a distinct viral lineage in the specific environment.

## Discussion

Intra-host variants were identified in many RNA viruses [22, 44, 45, 50–52]. Here, using deep sequencing data of serial samples, we revealed the existence of intra-host variation within COVID-19 patients. Given the observations in other RNA viruses [44, 46, 51], population bottleneck and purifying selection are the dominant factors driving the genetic differentiation during intra- and inter-evolutionary dynamics. Population bottlenecks could lead to a drastic reduction of viral population size during the early and middle stages of the epidemic [53–55]. Purifying selection becomes increasingly effective as the epidemic prolonged, because it has more opportunity to remove deleterious alleles [20, 56]. For SARS-CoV-2, one possible intra-host migration route is from the respiratory tract to the gastrointestinal epithelia. During the intra-host population bottlenecks, population composition may change dramatically through random sampling when a novel sub-population was established from a small group of viruses of a larger population [57]. This is supported by the clear genetic differentiation between RT and GIT populations. The stochastic process under neutral pressure plays an important role in intra-host population diversity during the early epidemic, as shown by the even distribution of AAFs among synonymous and non-synonymous iSNVs. Under this assumption, novel founder populations are expected to have a low genetic variation due to the subsampling from the original population. Following the bottleneck events, the viral genetic diversity of GIT populations might recover rapidly. This is also consistent with the high viral load in GIT. During viral replication, both RT and GIT populations generate intra-host variants.

Sharing of iSNVs usually could be derived from recurrent mutation, transmission, or contamination. We can rule out contamination, as seldom iSNVs are located at common SNP position. Observation of iSNV distributions among codon positions and genes suggests that the process of iSNV generation is probably site-independent. From the comparison of SNPs and iSNVs, SARS-CoV-2 genes have similar synonymous and non-synonymous patterns; most of the non-synonymous iSNVs could not be transmitted efficiently, suggesting selection is not the primary cause of shared iSNVs. Theoretically, selective pressures are inclined to be different for synonymous and non-synonymous mutations since most of non-synonymous mutations are deleterious. Besides we observed the possible fixation process, two iSNVs (G11083T and C21711T) were exclusively detected in the GIT populations of P01, P08, and P10. The fast fixation suggests that the resulting substitution might provide the virus certain selective advantages. Recently, similar research indicated that SARS-CoV-2 exhibits intra-host genomic plasticity and low-frequency polymorphic quasispecies [25, 26, 58]; the recurrent SARS-CoV-2 mutations currently in circulation appear to be evolutionary neutral [59]. Besides, the intra-host diversity of SARS-CoV-2 is frequently shared among infected individuals with patterns consistent with geographical structure [60, 61], which reflected the co-transmission of mixed population including shared SNP and iSNV, as the rapid geographic spread of SARS-CoV-2 in some countries. To assess the iSNVs detected in this study against other published data [26, 61], iSNV comparison was performed and interesting finding showed that seldom shared iSNVs were detected from different cohorts, which may be due to the geospatial constraints and individual discrepancy. However, most of iSNVs show a similar distribution pattern among genome, which were mainly scattered over ORF1ab, S, ORF3a, ORF6, ORF7, and N genes. Besides, seldom iSNV variants seem to arise many times along the phylogenetic tree, which may be derived from recurrent sequencing biases, hypermutability, or artifact [61–63]; the most remarkable example is G11083T, which is the most frequent and appears in both consensus sequences and iSNVs generated by multiple technologies. One hypothesis is that it appears to be due to a variable truncation of a long stretch of poly(T) at this position, which may present as a gap or one position immediately afterward depending on the analysis method [61]. On the other hand, the variant G11083T shows a strong phylogenetic signal, suggesting that they originated [63]. Related analyses or results should be caveated for the observation about the possible artifact.

In addition, our findings also demonstrated that those novel and/or recurrent variants are better accumulated and maintained within GIT, and hence, leading to a higher level of genetic diversity and potentially larger effective population size. In contrast, the intra-host variants are less stable in RT, probably associated with a more dramatic genetic drift in RT populations. In addition, viral adaptation against tissue-specific environment may also drive the divergence among intra-host populations, given the two GIT-specific non-synonymous iSNVs (G11083T and C21711T) observed in our study. SARS-CoV-2 needs to hijack the transcription and translational machinery of the host cell for a productive infection to happen. However, given the different susceptible cells, mechanisms of immune escape and microenvironment in RT and GIT, SARS-CoV-2 infecting different tissues may have adapted the viral variation and deleterious mutation removal towards their tropism. One plausible hypothesis is that the stronger selection pressure or immune response in the respiratory tract may restrict the population diversity of SARS-CoV-2, compared with the viral population in GIT. However, it is still challenging to fully disentangle the influences of stochastic processes and natural selection, considering the frequent confounding genetic signals of these two processes.

## Conclusions

Our results demonstrated a clear genetic differentiation between GIT and RT populations, mostly driven by bottleneck events among intra-host migrations. Compared to RT populations, the GIT populations showed a rapid accumulation and better maintenance of intra-host variants, reflecting a rapidly recovered genetic diversity following the intra-host migration. Nonetheless, given the presence of two GIT-specific non-synonymous iSNVs, tissue-specific adaptation may also drive the observed intra-host differentiation. Exact biological mechanisms of the intra-host population dynamics remain to be explored. Taken together, our data presented here illustrate the intra-host bottlenecks and evolution of SARS-CoV-2 and may provide new insights to understand the virus-host interactions of coronaviruses and other RNA viruses.

## Supplementary Information

**Additional file 1: Table S1.** Summary of clinical samples and patients with COVID-19. **Table S2.** List of public genomes used for analysis. **Table S3.** Allele frequency of iSNVs detected from metatranscriptomic and/or hybrid capture data**. Table S4.** Genomic information of 32 SARS-CoV-2 samples. **Table S5.** List of intra-host single nucleotide variants within 32 SARS-CoV-2 samples. **Table S6.** Genetic diversity of 32 SARS-CoV-2 samples. **Table S7.** Genetic distance between paired samples. **Table S8.** Frequency of proximal iSNVs using paired-end mapped reads.

**Additional file 2: Fig. S1.** Correlation of estimated alternative allele frequencies between metatranscriptomic and hybrid capture data. **Fig. S2.** Correlation of estimated minor alternative allele frequencies between biological replicates. **Fig. S3.** Correlation between sequencing depth and detected iSNVs. **Fig. S4.** Number of iSNV among three codon positions. **Fig. S5.** Haplotype frequency of proximal iSNVs within the gastrointestinal tract of patient P01.

## Data Availability

Sequence data have been deposited both in NCBI (https://www.ncbi.nlm.nih.gov/) under Project accession PRJNA698267 (https://www.ncbi.nlm.nih.gov/sra/PRJNA698267) [64] and in CNGB (https://db.cngb.org/) under Project accession CNP0000997 (https://db.cngb.org/search/project/CNP0000997/) [65] and CNP0001004 (https://db.cngb.org/search/project/CNP0001004/) [66]. The software used for data analyses in the current study are indicated in the manuscript.

## Abbreviations

SARS-CoV-2: Severe acute respiratory syndrome coronavirus 2
COVID-19: Coronavirus disease 2019
iSNVs: Intra-host single nucleotide variations
RT: Respiratory tract
GIT: Gastrointestinal tract
IAV: Influenza A virus
IBV: Influenza B virus
YFV: Yellow fever virus
GZMU: Guangzhou Medical University
AAFs: Alternative allele frequencies
ICU: Intensive care units
SNP: Single nucleotide polymorphisms
ORF: Open reading frame
