# Supplementary material for: Intra-host variation and evolutionary dynamics of SARS-CoV-2 populations in COVID-19 patients
Source: Genome Med. 2021 Feb 22;13:30. doi: 10.1186/s13073-021-00847-5 (PMC7898256; doi:10.1186/s13073-021-00847-5)
Supplement: Supplementary file 2 — Additional file 2: Fig. S1. Correlation of estimated alternative allele frequencies between metatranscriptomic and hybrid capture data. Fig. S2. Correlation of estimated minor alternative allele frequencies between biological replicates. Fig. S3. Correlation between sequencing depth and detected iSNVs. Fig. S4. Number of iSNV among three codon positions. Fig. S5. Haplotype frequency of proximal iSNVs within the gastrointestinal tract of patient P01. [file 13073_2021_847_MOESM2_ESM.pdf]

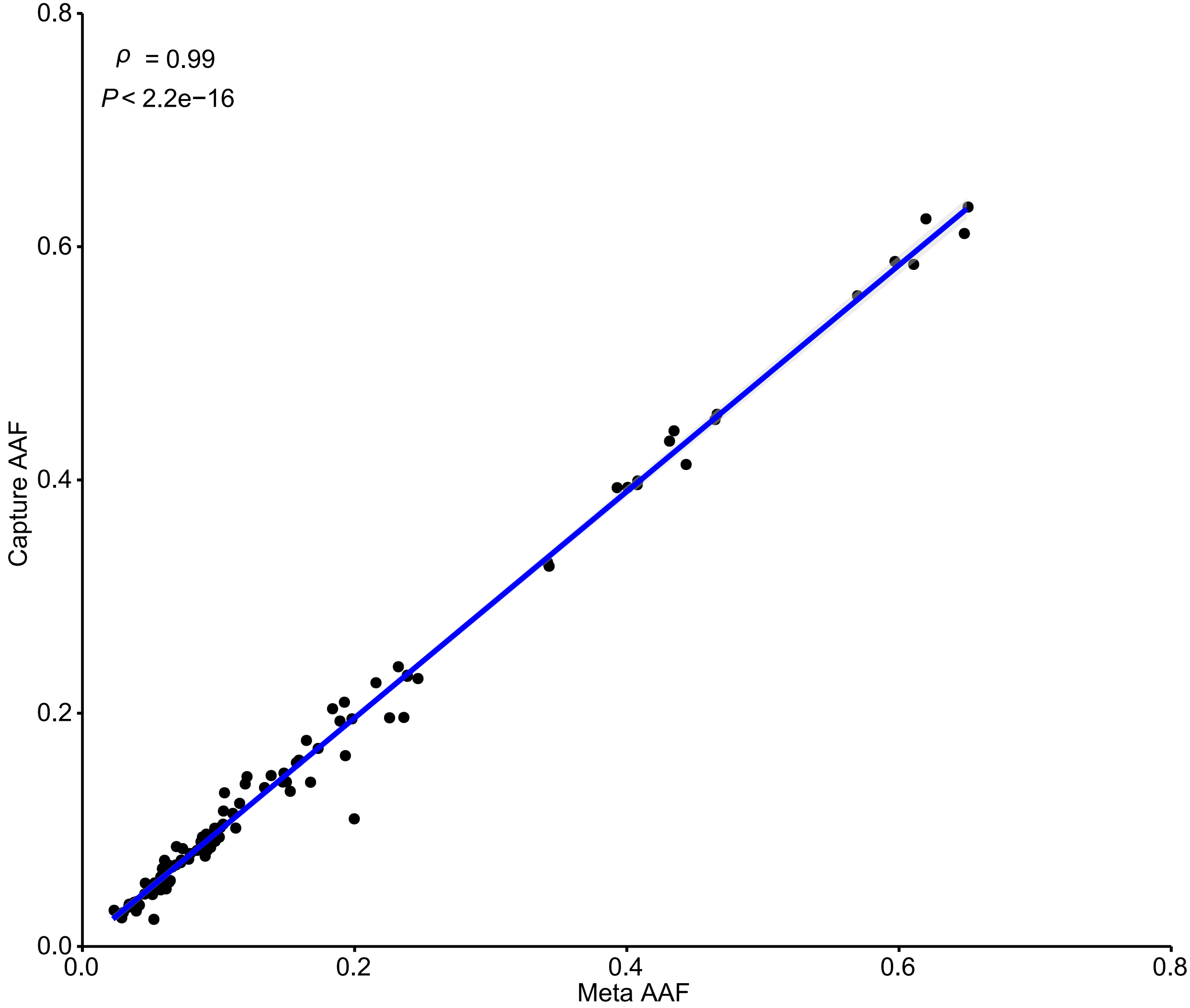

Fig. S1 Correlation of estimated alternative allele frequencies between metatranscriptomic and hybrid capture data.

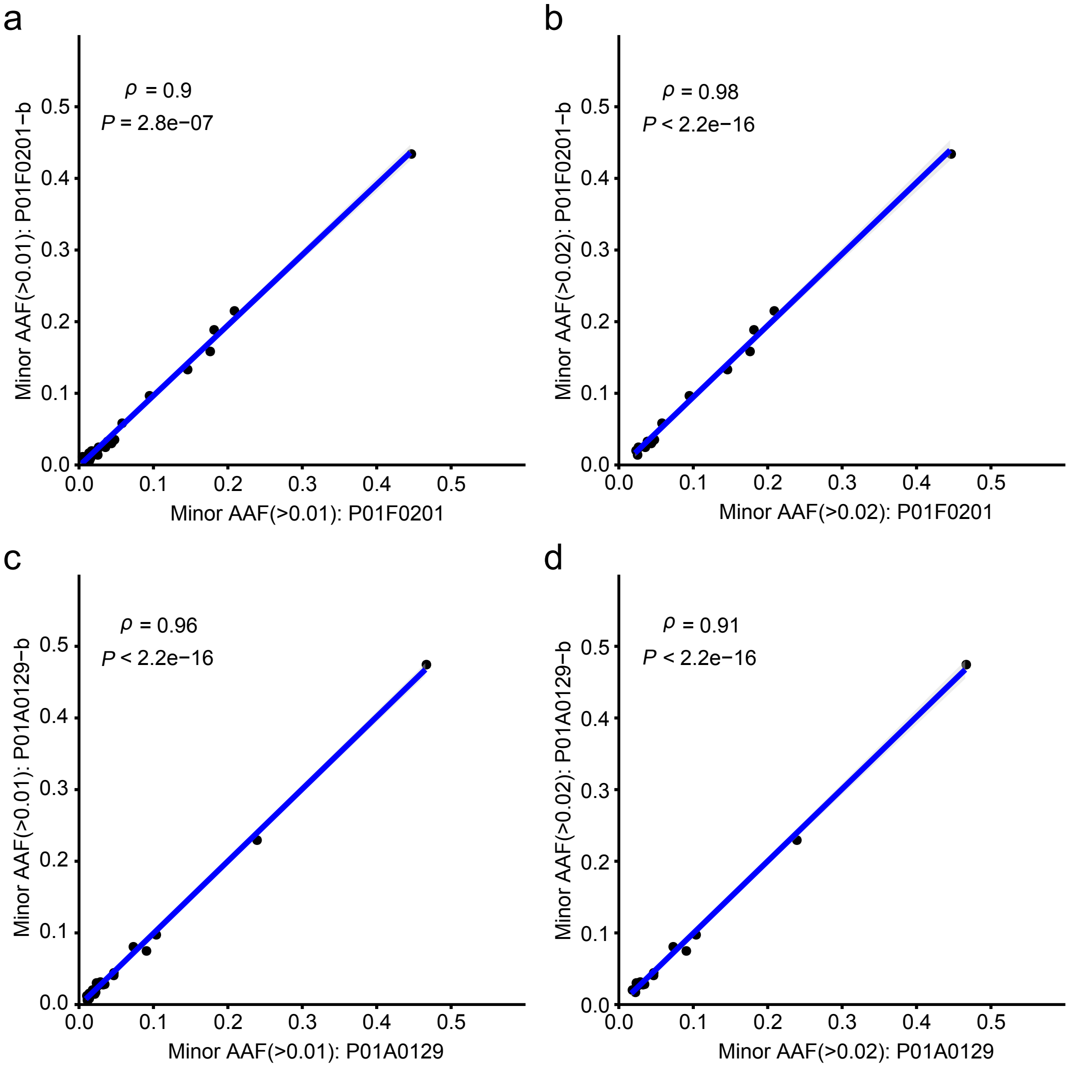

Fig. S2 Correlation of estimated minor alternative allele frequencies between biological replicates.

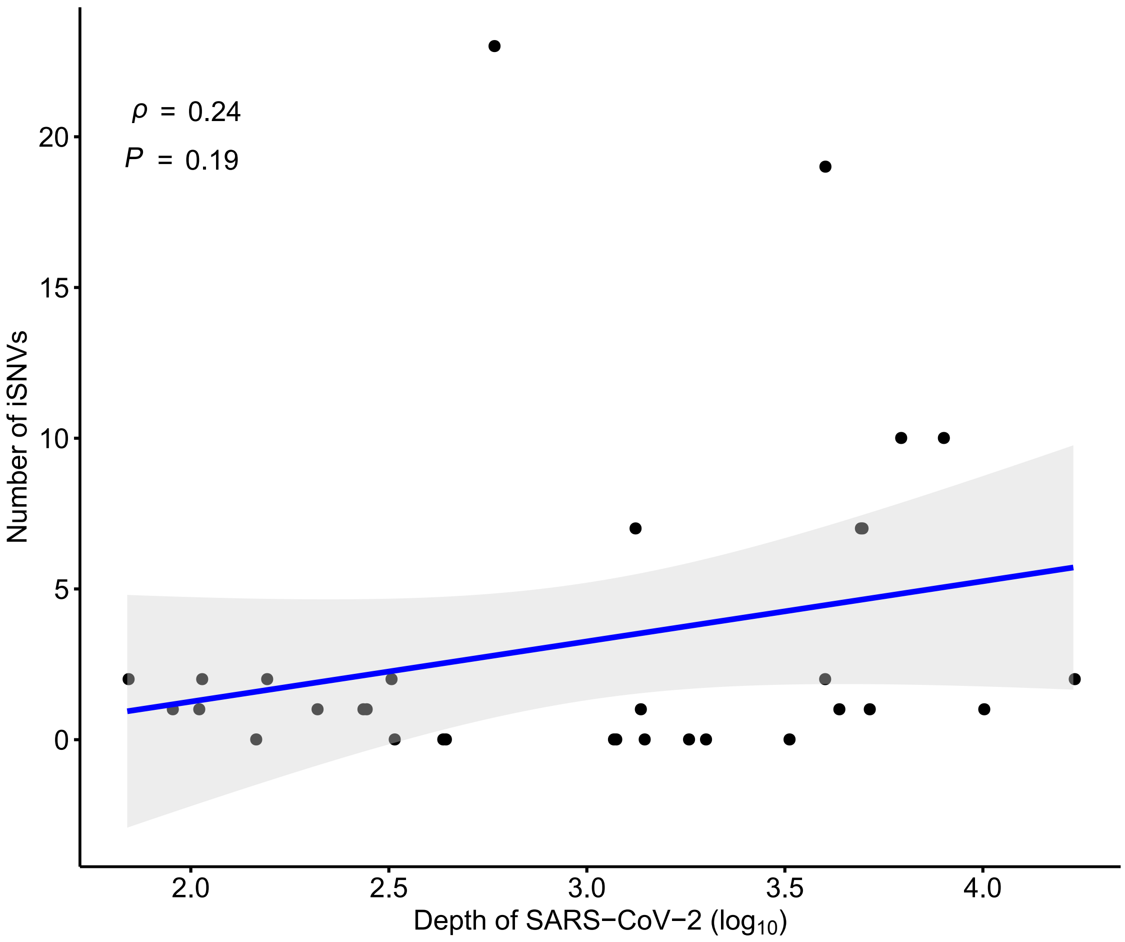

Fig. S3 Correlation between sequencing depth and detected iSNVs.

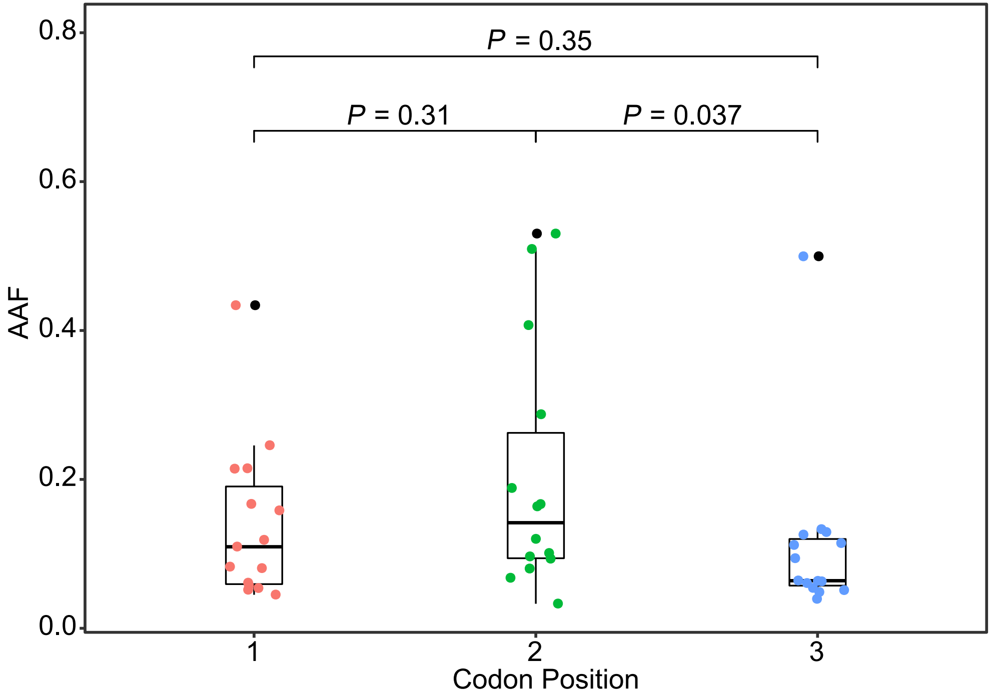

Fig. S4 Number of iSNV among three codon positions.

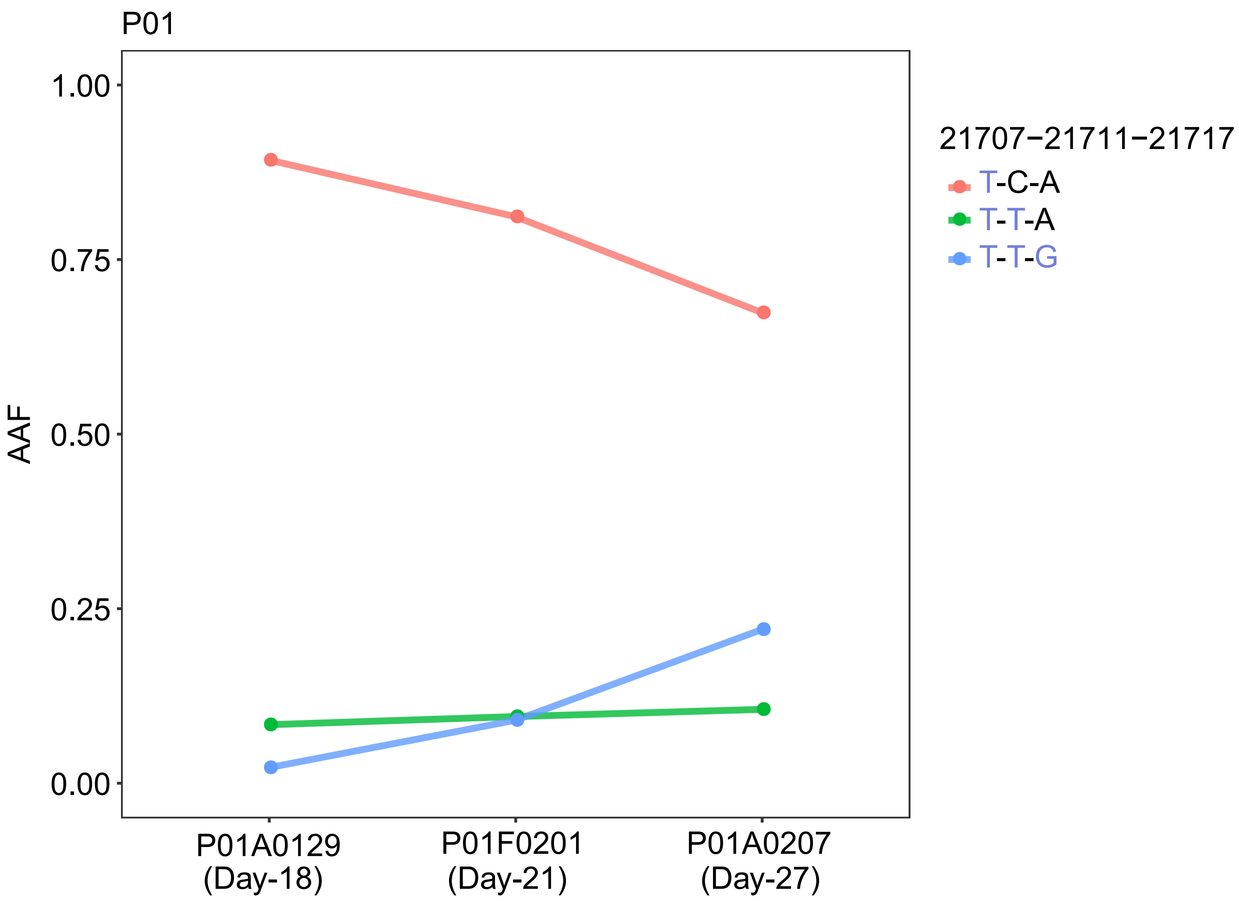

Fig. S5 Haplotype frequency of proximal iSNVs within the gastrointestinal tract of patient P01
